# Global Circumferential Strain by Cardiac Magnetic Resonance Tissue Tracking Associated With Ventricular Arrhythmias in Hypertrophic Cardiomyopathy Patients

**DOI:** 10.3389/fcvm.2021.670361

**Published:** 2021-05-28

**Authors:** Cailing Pu, Jingle Fei, Sangying Lv, Yan Wu, Chengbin He, Danling Guo, Pierre Umba Mabombo, Outesh Chooah, Hongjie Hu

**Affiliations:** ^1^Department of Radiology, Sir Run Run Shaw Hospital, Zhejiang University School of Medicine, Hangzhou, China; ^2^Department of Radiology, Shaoxing People's Hospital, Shaoxing, China

**Keywords:** cardiac magnetic resonance, hypertrophic cardiomyopathy, myocardial strain, tissue tracking, ventricular arrhythmias

## Abstract

**Background:** Hypertrophic cardiomyopathy (HCM) is prone to myocardial heterogeneity and fibrosis, which are the substrates of ventricular arrhythmias (VAs). Cardiac magnetic resonance tissue tracking (CMR-TT) can quantitatively reflect global and regional left ventricular strain from different directions. It is uncertain whether the change of myocardial strain detected by CMR-TT is associated with VAs. The aim of the study is to explore the differential diagnostic value of VAs in HCM by CMR-TT.

**Materials and Methods:** We retrospectively included 93 HCM patients (38 with VAs and 55 without VAs) and 30 healthy cases. Left ventricular function, myocardial strain parameters and percentage of late gadolinium enhancement (%LGE) were evaluated.

**Results:** Global circumferential strain (GCS) and %LGE correlated moderately (*r* = 0.51, *P* < 0.001). HCM patients with VAs had lower left ventricular ejection fraction (LVEF), global radial strain (GRS), GCS, and global longitudinal strain (GLS), but increased %LGE compared with those without VAs (*P* < 0.01 for all). %LGE and GCS were indicators of VAs in HCM patients by multivariate logistic regression analysis. HCM patients with %LGE >5.35% (AUC 0.81, 95% CI 0.70–0.91, *P* < 0.001) or GCS >-14.73% (AUC 0.79, 95% CI 0.70–0.89, *P* < 0.001) on CMR more frequently had VAs. %LGE + GCS were able to better identify HCM patients with VAs (AUC 0.87, 95% CI 0.79–0.95, *P* < 0.001).

**Conclusion:** GCS and %LGE were independent risk indicators of VAs in HCM. GCS is expected to be a good potential predictor in identifying HCM patients with VAs, which may provide important values to improve risk stratification in HCM in clinical practice.

## Introduction

Hypertrophic cardiomyopathy (HCM) is the most common hereditary cardiomyopathy with a prevalence of 1 per 500 persons in the world (1). Histological findings in HCM include hypertrophic cardiomyocytes, myocardial disarray, and fibrosis (2, 3). The clinical manifestations of HCM patients are diverse. Sudden cardiac death (SCD) is the most severe complication in HCM patients, and the prediction of SCD is always a challenge for clinicians (4). Some studies showed that ventricular arrhythmias (VAs) were responsible for SCD in HCM patients. The increased heterogeneity of myocardial conduction, which is the substrate for the formation of VAs, seems to be associated with myocardial fibrosis in HCM patients with VAs (5).

Cardiac magnetic resonance (CMR) is an excellent diagnostic imaging modality to assess the structure and function of heart disease (6–8). It has been reported that late gadolinium enhancement (LGE) in CMR can reflect myocardial fibrosis, and the size of fibrosis detected by LGE can predict the occurrence of VAs and SCD (4, 9). However, LGE is contraindicated in patients with severe impaired renal function. Left ventricular function parameters, such as left ventricular ejection fraction (LVEF), left ventricular end-diastolic volume index (LVEDVI), and stroke volume index (SVI) can reflect the pumping function of the heart. Nevertheless, the aforementioned parameters are often in the normal range or even increased because of the compensatory effect of HCM (10), hence, unable to accurately assess the abnormal myocardial function. Therefore, we tried to use a new method to evaluate the myocardial motion in HCM and to explore the correlation between myocardial strain and VAs.

Recently, myocardial strain detected by CMR tissue tracking (CMR-TT) has been widely used in clinical studies to estimate systolic or diastolic dysfunction in cardiac diseases, such as coronary artery disease, dilated cardiomyopathy, hypertrophic cardiomyopathy, and restrictive cardiomyopathy (11–13). CMR-TT can quantitatively measure myocardial strain from radial, circumferential, and longitudinal directions based on the cine views, which can accurately reflect the global and regional left ventricular function (11). It has also been shown that myocardial strain parameters have some correlation with myocardial hypertrophy, myocardial fibrosis, and microcirculation perfusion disorder (14–16). Therefore, this study aimed to evaluate myocardial strain of HCM patients by CMR-TT, and analyze the correlation between myocardial strain and the extent of myocardial fibrosis and further to explore the value of myocardial strain parameters in identifying whether HCM patients with VAs or without VAs.

## Materials and Methods

### Study Population

**Figure 1:**
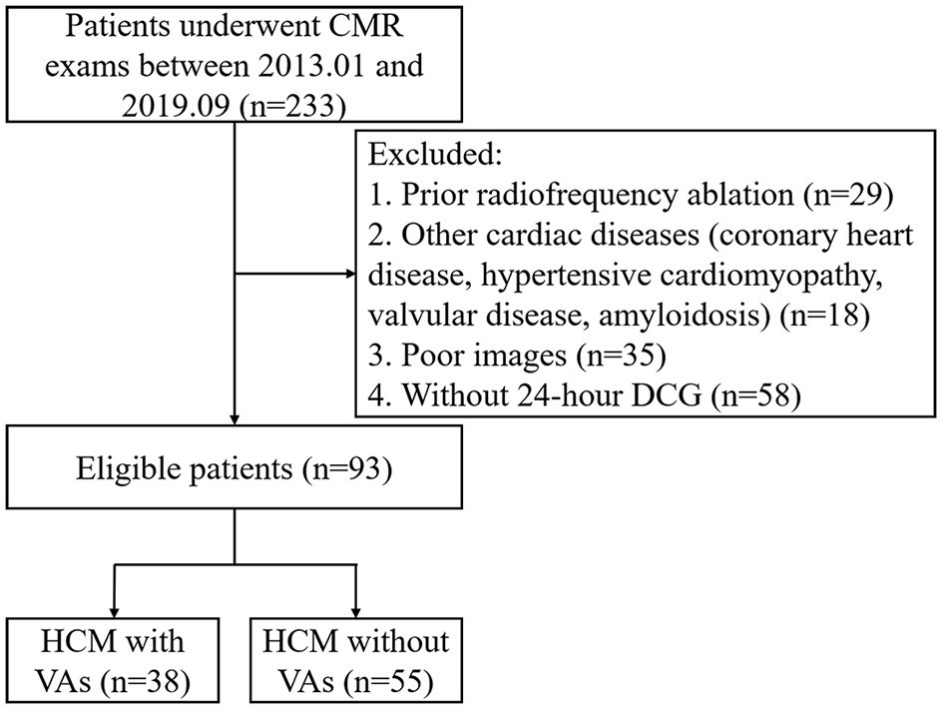
Flow diagram showing the selection and group of HCM patients. CMR, cardiac magnetic resonance; DCG, dynamic electrocardiogram; HCM, hypertrophic cardiomyopathy; VA, ventricular arrhythmia.

This retrospective study complied with the Declaration of Helsinki and was approved by our institutional ethics committee. The requirement for written informed consent was waived because of the retrospective nature of the study. From January 2013 to September 2019, 93 adult patients with HCM and 30 healthy control subjects were included in this study (Figure 1). Inclusion criteria of HCM patients were determined by the maximal left ventricle thickness on CMR images ≥15 mm, or ≥13 mm with a documented family history of HCM (17). Major exclusion criteria were as follows: previous radiofrequency or alcohol ablation, poor or incomplete images, without 24-h dynamic electrocardiogram (DCG), and coronary artery disease and myocardial hypertrophy of other causes, e.g., valvular disease, hypertensive cardiomyopathy or poorly controlled hypertension, and cardiac amyloidosis. All HCM patients underwent clinical examination, CMR, and 24-h DCG. According to 24-h DCG, HCM patients were divided into HCM with VAs group (38 cases) and HCM without VAs group (55 cases). The presence of VAs was defined as previous aborted cardiac arrest, documented sustained ventricular tachycardia (VT: defined as sustained ventricular arrhythmia over 100 heartbeats per minute when lasting longer than 30 s or requiring earlier intervention due to hemodynamic instability), and non-sustained VT (NSVT: defined as an episode ≥3 beats with >100 heartbeats per minute, and a maximum episode length of 30 s during 24-h DCG) (18). The exclusion criteria of control subjects were combined with chronic diseases, family history of cardiac disease, hypertension, and arrhythmias.

### Cardiac Magnetic Resonance

All CMR views were performed on 1.5-T magnetic resonance (MR) scanners (GE MR Signa HD excite, America or Siemens Magnetom Avanto, Germany) with a phased array body coil. Short-axis cine imaging covering the whole left ventricle and long-axis cine imaging (two/three/four-chamber) were performed using an ECG-gated, breath-hold, and balanced steady-state free precession sequence with the following parameters: 8 mm slice thickness with 2 mm interslice gap, repetition time 3.5/47.52 ms, echo time 1.5/1.11 ms, flip angle of 45/56°, and field of view 360/340 mm. LGE imaging was obtained 8–10 min after intravenous injection of 0.2 mmol/kg of gadopentetate dimeglumine (Beilu, Beijing, China) with the following parameters: 8 mm slice thickness with 2 mm interslice gap, repetition time 6.5/711 ms, echo time 3.5/1.09 ms, flip angle of 20/40°, and field of view 360/340 mm.

### Imaging Analysis

**Figure 2:**
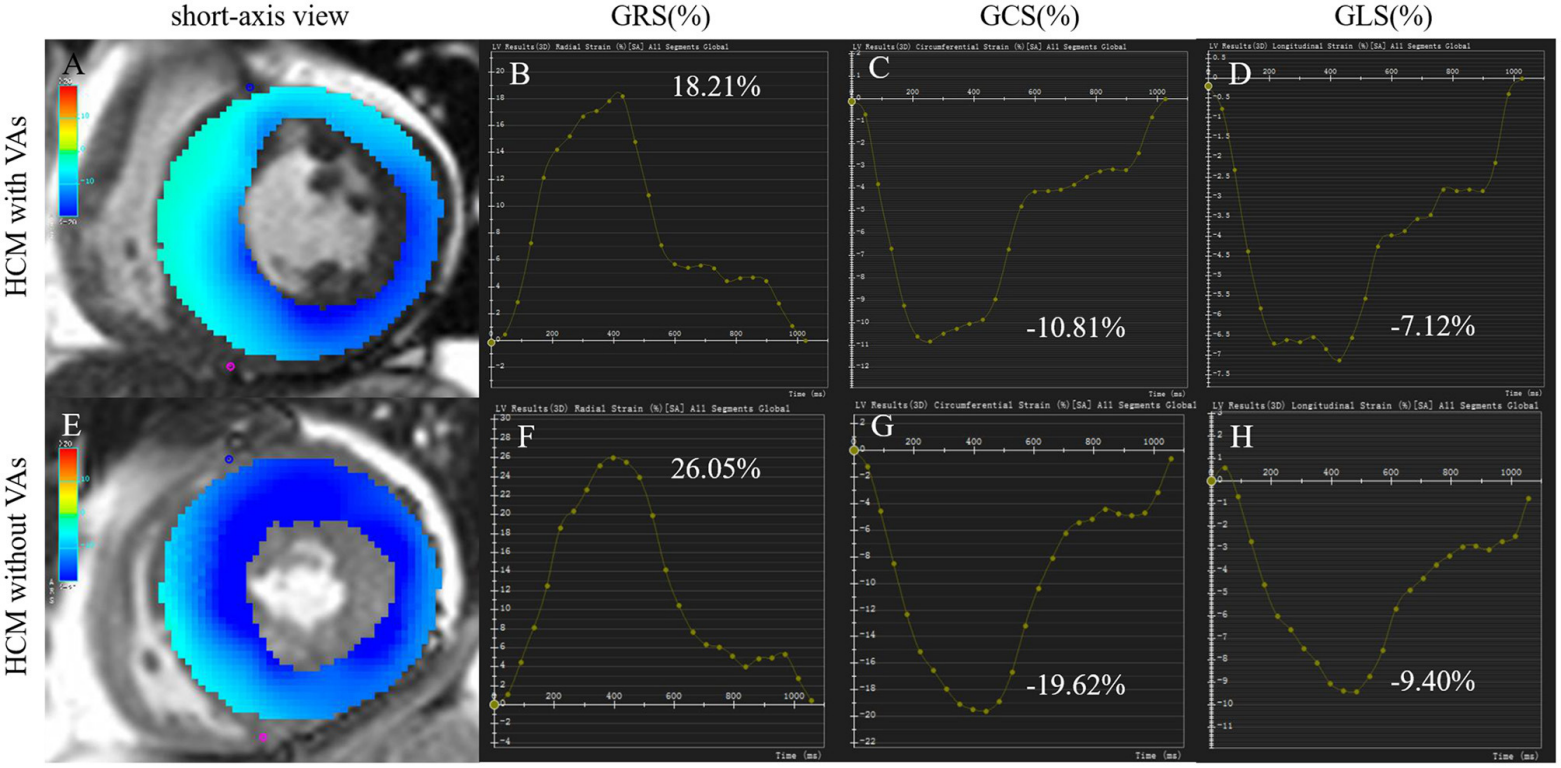
Tissue tracking and strain analysis in HCM with and without VAs. Left column **(A,E)**: color overlap map of short-axis view. Right columns **(B–D, F–H)**: strain analysis curves of GRS, GCS, and GLS. The myocardial strain parameters in a 55-year-old man with VAs **(A–D)** were much lower than those in a 74-year-old woman without VAs **(E–H)**. GCS, global circumferential strain; GLS, global longitudinal strain; GRS, global radial strain; HCM, hypertrophic cardiomyopathy; VA, ventricular arrhythmia.

CMR imaging analysis was performed with commercially available software (Circle Cardiovascular Imaging 42, version 5.10.1, Calgary, Canada). Left ventricular function, including LVEF, LVEDVI, left ventricular end-systolic volume index (LVESVI), SVI, and left ventricular mass index (LVMI), were generated automatically by loading short-axis imaging into the short-3D module. Maximum wall thickness (MWT) was measured at the end of diastole in short-axis imaging. Left atrial anteroposterior diameter (LAD-AP) was measured on three-chamber view. A set of long-axis (two/three/four-chamber) and short-axis views were loaded into the tissue tracking module to analyze myocardial strain, which referred to the degree of myocardial deformation from its initial length (L_0_, usually in the end diastole) to its maximum length (L, usually in the end systole) and was expressed as a percentage: (L – L_0_)/L × 100%, including global radial strain (GRS), global circumferential strain (GCS), global longitudinal strain (GLS), or rate of shortening of the length (1/s), such as global radial strain of diastolic rate (GRSDr), global circumferential strain of diastolic rate (GCSDr), and global longitudinal strain of diastolic rate (GLSDr). Radial strain was expressed by positive value which indicated the myocardium thickening and thinning motion toward the center of the cavity in the radial direction. Circumferential strain was expressed by negative value which derived from myocardium shortening along the circular perimeter observed on a short-axis view. Longitudinal strain was expressed by negative value which represented the longitudinal shortening from the base to the apex. Examples of tissue tracking and strain analysis in HCM with and without VAs are described in Figure 2. Identified LGE was defined using the grayscale threshold over 5 SDs from remote normal myocardium in the tissue characteristic module (19), and areas of LGE were quantified as the percentage of total left ventricular mass (%LGE).

In some HCM patients without left ventricular outflow tract (LVOT) obstruction, exact values of LVOT pressure gradient were not documented. Therefore, we only calculated SCD score in 56 HCM patients with recorded values of LVOT pressure gradient, 23 with VAs and 33 without VAs.

### Inter- and Intra-observer Reproducibility

To assess inter-observer reproducibility, images of 10 randomly selected HCM patients (five patients with VAs and five patients without VAs) and 10 controls were independently analyzed by two radiologists with more than 2-year experience. For intra-observer reproducibility, one radiologist reanalyzed the images of the same 20 participants.

### Statistical Analysis

Continuous and normal variables were represented by means ± SD, while data that did not fit the normal distribution were described by median and quartile (interquartile range). Differences between the two groups were evaluated by independent *t*-test or Wilcoxon signed-rank test. Categorical variables were expressed as frequency (percentage) and assessed using χ^2^ test or Fisher's exact test. Correlation of non-normal distribution variables were evaluated using Spearman rank correlation coefficient. Univariate logistic regression was used to identify markers for VAs in HCM. Multivariate analysis was significantly performed (*P* < 0.05) using variables from the univariate analysis. Receiver operator characteristic (ROC) curves were used to determine the area under the curve (AUC), CI, and optimal sensitivity and specificity to discriminate HCM patients with VAs. The optimal cut-off values were determined by calculating Youden index. Inter- and intra-observer variabilities for %LGE and strain were assessed using the intraclass correlation coefficient (ICC). Two-tailed values of *P* < 0.05 were considered statistically significant. All data were analyzed using SPSS (version 23.0; IBM, Armonk, NY, USA) and GraphPad Prism (version 8.3.0; GraphPad Software Inc., San Diego, CA, USA).

## Results

### Participant Characteristics

The demographics, left cardiac function, and myocardial strain data are summarized in Table 1. We included 93 HCM patients and their mean age was 54 ± 14 years, and 63 (68%) patients were men. HCM patients were associated with higher LVEF, SVI, LVMI, MWT, and LAD-AP compared with the control subjects (*P* < 0.001 for all), but lower LVESVI, GRS, GCS, GLS, GRSDr, GCSDr, and GLSDr (*P* ≤ 0.001 for all).

**Table 1:** The baseline characteristics of all participants.

	**Control (*n* = 30)**	**HCM (*n* = 93)**	***P*-value**
**Clinical data**			
Age (years)	47 ± 15	54 ± 14	0.03
Male, n (%)	16 (53)	63 (68)	0.15
Weight (kg)	64 ± 11	67 ± 11	0.26
Height (cm)	167 (162, 173)	166 (160, 172)	0.30
BSA (m^2^)	1.72 ± 0.17	1.75 ± 0.18	0.49
**Cardiac function**			
LVEF (%)	61.99 (57.41, 65.00)	71.37 (63.10, 76.97)	<0.001
LVEDVI (ml/m^2^)	75.21 (68.22, 84.05)	73.60 (66.48, 86.66)	0.70
LVESVI (ml/m^2^)	28.28 (24.85, 33.16)	20.84 (16.19, 28.40)	<0.001
SVI (ml/m^2^)	46.33 (42.82, 51.32)	51.63 (43.44, 55.99)	0.01
LVMI (g/m^2^)	50.80 (47.84, 57.55)	85.99 (69.44, 101.11)	<0.001
**Morphologic data**			
MWT (mm)	9 (8,10)	21 (18,26)	<0.001
LAD-AP (mm)	32 (29, 34)	39 (35, 45)	<0.001
LGE present, *n* (%)	0 (0)	79 (85)	–
**Myocardial strain**			
GRS (%)	31.77 (28.05, 36.99)	23.20 (17.25, 35.67)	0.001
GCS (%)	−19.41 ± 3.40	−16.93 ± 4.73	<0.01
GLS (%)	−12.73 (−14.92, −11.02)	−8.73 (−11.48, −6.75)	<0.001
GRSDr (1/s)	−2.43 (−3.09, −1.79)	−1.34 (−2.18, −0.89)	<0.001
GCSDr (1/s)	1.13 (0.95, 1.46)	0.87 (0.64, 1.09)	<0.001
GLSDr (1/s)	0.71 (0.52, 0.98)	0.51 (0.40, 0.63)	<0.001

*Data are presented as mean (SD) or absolute number (percentage) or median (interquartile range). BSA, body mass index; GCS, global circumferential strain; GCSDr, global circumferential strain of diastolic rate; GLS, global longitudinal strain; GLSDr, global longitudinal strain of diastolic rate; GRS, global radial strain; GRSDr, global radial strain of diastolic rate; HCM, hypertrophic cardiomyopathy; LAD-AP, left atrial anteroposterior diameter; LGE, late gadolinium enhancement; LVEDVI, left ventricular end-diastolic volume index; LVEF, left ventricular ejection fraction; LVESVI, left ventricular end-systolic volume index; LVMI, left ventricular mass index; MWT, maximum wall thickness; SVI, stroke volume index*.

In HCM with VAs, 11 patients received ICD and 3 of them underwent discharge therapy. Another patient developed ventricular fibrillation during hospitalization. In HCM without VAs, only one patient received ICD for primary prevention (Table 2). In HCM with VAs, 5-year SCD risk score was much higher than those without VAs [5.45 (3.88, 6.36) vs. 1.80 (1.45, 2.23), *P* < 0.001; Table 2].

**Table 2:** Clinical characteristics and CMR findings in HCM patients with and without VAs.

	**HCM with VAs (*n* = 38)**	**HCM without VAs (*n* = 55)**	***P*-value**
**Clinical data**			
Age (years)	56 ± 13	53 ± 15	0.20
Male, *n* (%)	25 (66)	38 (69)	0.74
Weight (kg)	166 (160, 170)	168 (160, 173)	0.62
Height (cm)	66 ± 10	67 ± 12	0.73
BSA (m^2^)	1.74 ± 0.16	1.75 ± 0.18	0.75
Family history, *n* (%)	4 (11)	5 (9)	0.82
Syncope, *n* (%)	8 (21)	6 (11)	0.18
Hypertension, *n* (%)	17 (45)	23 (42)	0.78
Diabetes, *n* (%)	4 (11)	4 (7)	0.71
ICD, n (%)	11 (29)	1 (2)	<0.001
SCD score*	5.45 (3.88, 6.36)	1.80 (1.45, 2.23)	<0.001
**Medications**			
Amiodarone, *n* (%)	3 (8)	1 (2)	0.30
Beta-blocker, *n* (%)	24 (63)	38 (69)	0.55
Calcium channel blocker, *n* (%)	10 (26)	19 (35)	0.40
ACEI, *n* (%)	4 (11)	3 (5)	0.44
ARB, *n* (%)	7 (18)	13 (24)	0.55
Statins, *n* (%)	14 (37)	18 (33)	0.68
Diuretic, *n* (%)	3 (8)	3 (5)	0.69
Aldosterone antagonist, *n* (%)	3 (8)	2 (4)	0.40
**Cardiac function**			
LVEF (ml/m^2^)	65.16 (55.16, 74.41)	73.51 (68.50, 77.63)	<0.01
LVEDVI (ml/m^2^)	79.92 (69.78, 91.45)	69.47 (65.36, 79.32)	<0.01
LVESVI (ml/m^2^)	26.07 (18.06, 43.32)	19.82 (15.16, 24.43)	<0.01
SVI (ml/m^2^)	51.50 (42.17, 54.83)	51.96 (44.96, 57.16)	0.29
LVMI (g/m^2^)	95.62 (82.51, 123.73)	78.05 (67.41, 95.57)	0.001
**Morphologic data**			
Obstruction present, *n* (%)	15 (39)	19 (35)	0.63
MWT (mm)	24 (19,28)	20 (18,23)	<0.01
LAD-AP (mm)	42 ± 7	38 ± 7	<0.01
LGE present, *n* (%)	33 (87)	45 (82)	0.52
%LGE	9.70 (5.76, 20.32)	2.29 (1.02, 4.08)	<0.001
**Myocardial strain**			
GRS (%)	17.72 (12.62, 24.19)	27.31 (20.07, 37.65)	<0.001
GCS (%)	−14.11 ± 4.47	−18.88 ± 3.86	<0.001
GLS (%)	−7.35 ± 3.50	−10.23 ± 2.92	<0.001
GRSDr (1/s)	−1.05 (−1.48, −0.70)	−1.45 (−2.44, −1.15)	0.001
GCSDr (1/s)	0.66 (0.54, 0.90)	0.98 (0.80, 1.15)	<0.001
GLSDr (1/s)	0.47 (0.33, 0.54)	0.57 (0.47, 0.69)	<0.01

Data are presented as mean (SD) or absolute number (percentage) or median (interquartile range). *Not all HCM patients calculated SCD score. ACEI, angiotensin converting enzyme inhibitor; ARB, angiotensin receptor blocker; CMR, cardiac magnetic resonance; ICD, implantable cardioverter-defibrillator; SCD, sudden cardiac death; VA, ventricular arrhythmia (others, see Table 1).

### LGE and Myocardial Strain in HCM Patients

**Figure 3:**
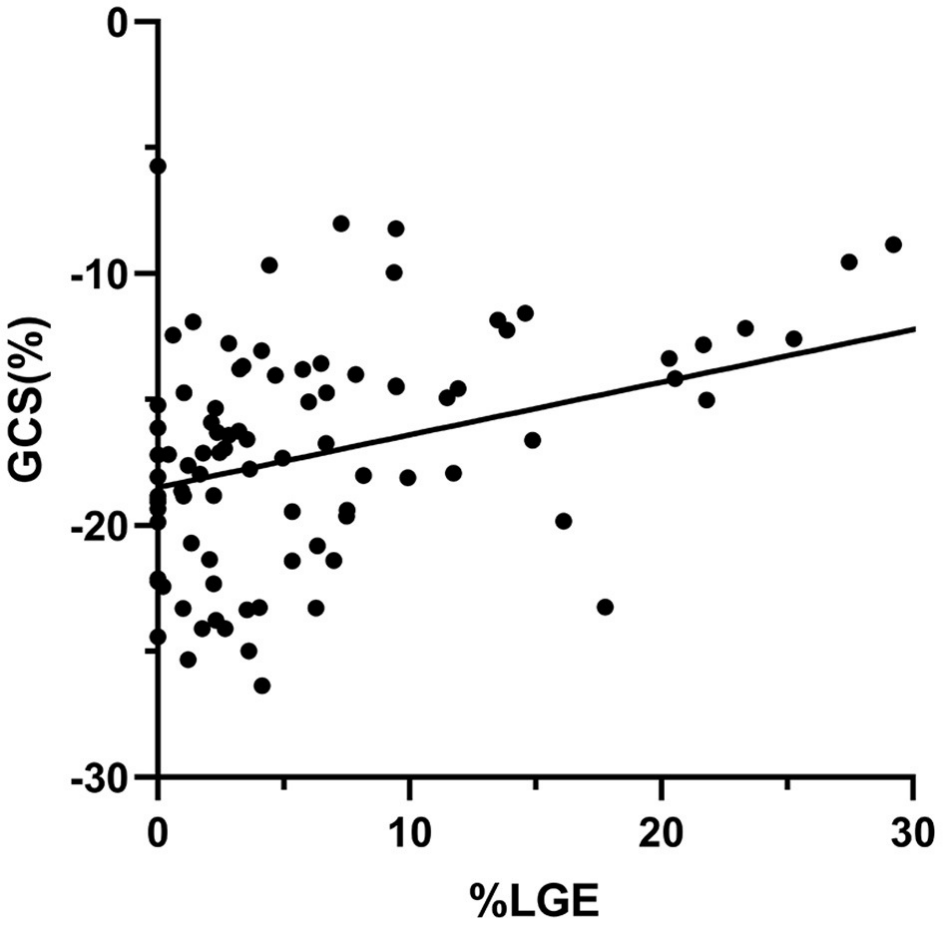
Scatter plots of correlation analysis between GCS and %LGE. GCS and %LGE were correlated moderately (*r* = 0.51, *P* < 0.001). GCS, global circumferential strain; LGE, late gadolinium enhancement.

A total of 78 (84%) HCM patients had LGE present. Patients with LGE had a tendency toward worse GRS, GCS, GLS [21.28 (16.86, 34.08) vs. 27.96 (24.45, 37.36), *P* = 0.06; −16.49 (−19.82, −13.56) vs. −18.96 (−19.86, −17.40), *P* = 0.08; −8.36 (−11.22, −6.65) vs. −10.47 (−12.12, −8.34), *P* = 0.07] compared with those without LGE (Supplementary Table 1). By Spearman rank correlation coefficient, GCS and %LGE were correlated moderately (*r* = 0.51, *P* < 0.001; Figure 3). There was a weak but significant correlation between %LGE and GRS, GLS, and GCSDr (*r* = −0.38, *P* < 0.001; *r* = 0.34, *P* = 0.001; *r* = −0.31, *P* < 0.01). No correlation was found between %LGE and GRSDr and GLSDr (Supplementary Table 2).

### CMR Findings Related to VAs in HCM Patients

**Figure 4:**
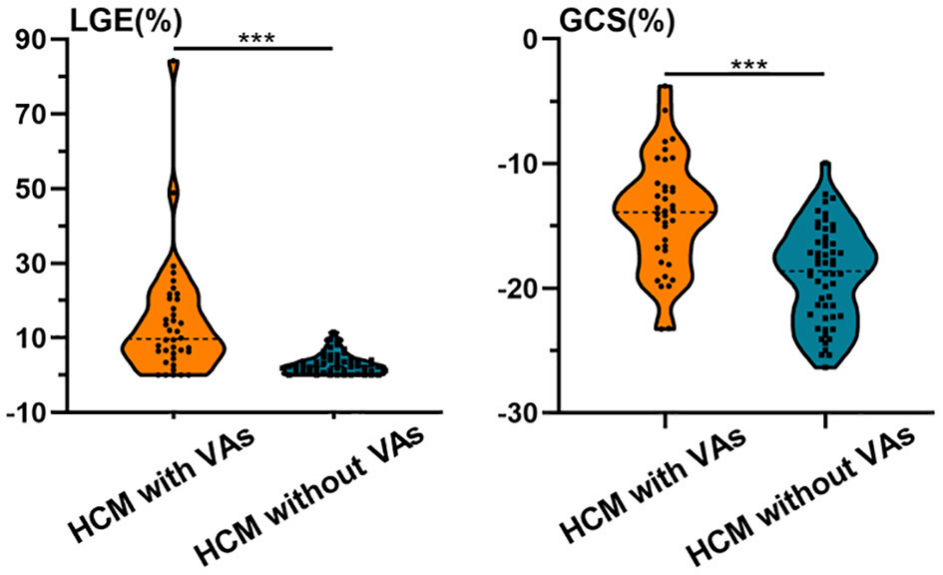
Violin plots of %LGE and GCS in HCM patients with and without VAs. HCM patients with VAs had worse GCS and increased %LGE compared with those without VAs. Asterisks denote *P* < 0.001. GCS, global circumferential strain; HCM, hypertrophic cardiomyopathy; LGE, late gadolinium enhancement; VA, ventricular arrhythmia.

**Figure 5:**
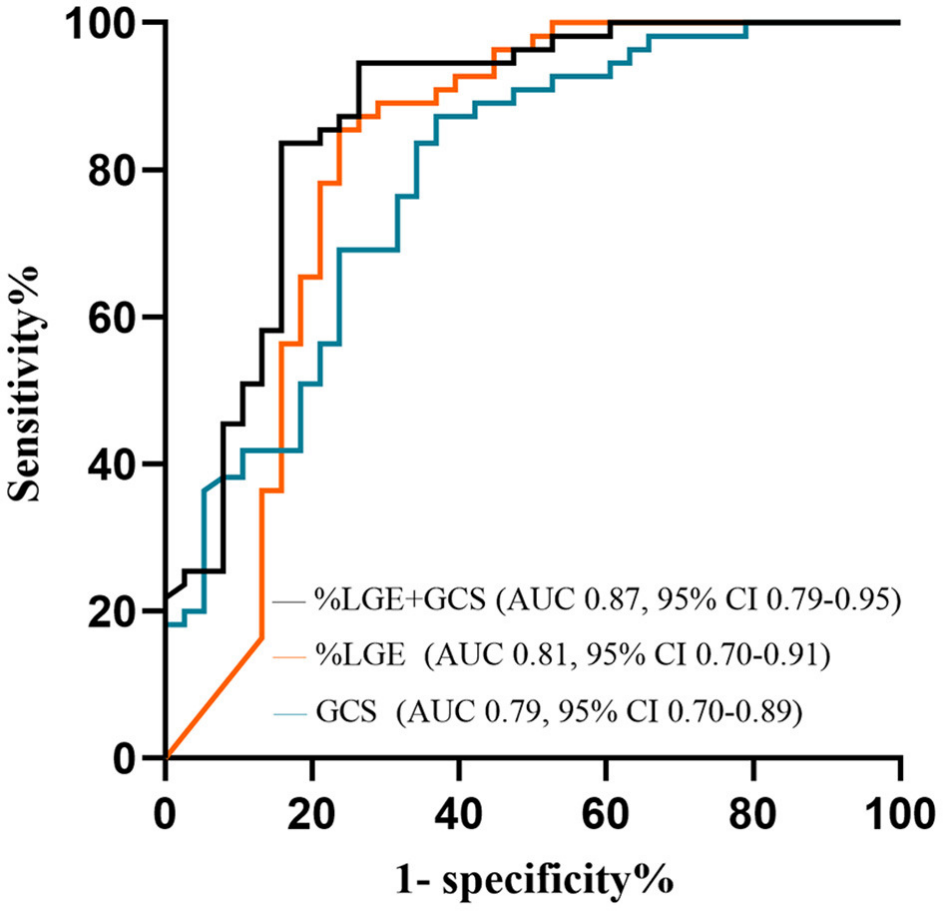
ROC curves to assess the ability of %LGE and GCS in identifying HCM patients with VAs. AUC, area under the curve; GCS, global circumferential strain; HCM, hypertrophic cardiomyopathy; LGE, late gadolinium enhancement; ROC, receiver operator characteristic; VA, ventricular arrhythmia.

HCM patients with VAs had worse LVEF and myocardial strain, higher LVEDVI, and LVESVI, thicker MWT, and increased %LGE compared with those without VAs (*P* < 0.01 for all; Table 2 and Figure 4). Further multivariate logistic regression analysis showed that %LGE and GCS were markers of VAs in HCM patients after adjusting beta-blocker use (Table 3). Using optimal cut-off values from ROC curves, HCM patients with %LGE >5.35% [AUC 0.81, 95% CI (0.70, 0.91), *P* < 0.001] or GCS >-14.73% [AUC 0.79, 95% CI (0.70, 0.89), *P* < 0.001] on CMR more frequently had VAs, and there was no significant difference between the two indicators by DeLong's test (*P* = 0.75). When combining %LGE with GCS to diagnose HCM with VAs, AUC was 0.87 [95% CI (0.79, 0.95), *P* < 0.001], which was better than the diagnostic performance of GCS (*P* = 0.04) and tended to be better than that of %LGE (*P* = 0.06; Figure 5).

**Table 3:** Univariate and multivariate logistic regression analysis for predictors of VAs in HCM patients.

	**Univariate logistic regression**		**Multivariate logistic regression**	
	**OR (95% CI)**	***P-*value**	**OR (95% CI)**	***P*-value**
Age (years)	1.02 (0.99, 1.05)	0.20		
Male, *n* (%)	1.16 (0.48, 2.81)	0.74		
Beta-blocker, *n* (%)	0.77 (0.32, 1.84)	0.55		
LVEF (ml/m^2^)	0.94 (0.90, 0.98)	< 0.01		
LVEDVI (ml/m^2^)	1.04 (1.01, 1.07)	< 0.01		
LVESVI (ml/m^2^)	1.07 (1.02, 1.11)	< 0.01		
SVI (ml/m^2^)	0.99 (0.95, 1.02)	0.44		
LVMI (g/m^2^)	1.03 (1.01, 1.05)	< 0.01		
Obstruction present, n (%)	1.24 (0.53, 2.91)	0.63		
MWT (mm)	1.14 (1.04, 1.24)	< 0.01		
LAD-AP (mm)	1.09 (1.02, 1.17)	< 0.01		
LGE present, *n* (%)	1.47 (0.46, 4.70)	0.52		
%LGE	1.28 (1.14, 1.44)	< 0.001	1.24 (1.10, 1.40)	< 0.001
GRS (%)	0.93 (0.90, 0.97)	0.001		
GCS (%)	1.33 (1.16, 1.52)	< 0.001	1.22 (1.06, 1.41)	< 0.01
GLS (%)	1.36 (1.15, 1.60)	< 0.001		
GRSDr (1/s)	1.18 (0.84, 1.66)	0.35		
GCSDr (1/s)	0.13 (0.03, 0.55)	< 0.01		
GLSDr (1/s)	0.06 (0.01, 0.57)	< 0.05		

*Data are presented as OR (95% CI). Abbreviations: see Table 1*.

### Intra- and Inter-observer Reproducibility

We found good reproducibility of %LGE and all strain measurements for intra- and inter-observer variability (ICC values for GCS were over 0.9). Summaries of the ICC values in both intra- and inter-observer reproducibility are shown in Supplementary Table 3.

## Discussion

Our study demonstrated impaired left ventricular global strain in the radial, circumferential, and longitudinal directions in HCM patients using CMR-TT. We found a correlation between myocardial strain and fibrosis confirmed by LGE in HCM patients. More importantly, GCS and %LGE were demonstrated to be strong and independent predictors of VAs.

### Left Ventricular Function in HCM

In this study, we found that LVEF and SVI in HCM patients were significantly increased. However, their LVESVI, GRS, GCS, and GLS were obviously decreased, indicating early myocardial strain damage compared with LVEF and SVI in HCM patients. It was mainly explained by the fact that hypertrophic myocardium pathology induced hyperejection status led to normal or even higher LVEF and SVI (10). In fact, the disordered arrangement of hypertrophic cardiomyocytes and fibers caused sarcomeric systolic dysfunction. Myocardial strain could reflect more accurately systolic function and was not affected by global movement and adjacent myocardium (10, 11). Therefore, myocardial strain has already reduced at an early stage in HCM patients. Furthermore, observed myocardial diastolic strain rates (GRSDr, GCSDr, GLSDr) were much lower than healthy control group, while LVEDVI was almost normal. This result strengthened and proved the sensitivity of myocardial strain parameters in the evaluation of myocardial diastolic dysfunction.

### VAs and Myocardial Strain

This study demonstrated that reduced GCS and increased %LGE were markers of VAs on 24-h DCG in HCM patients. GCS and %LGE correlated moderately (*r* = 0.51, *P* < 0.001). With the progression of HCM, disarrangement of myocardial fiber bundles and extent of fibrotic myocardium raised gradually (5). As a result, aggravation of myocardium heterogeneity became severe, which formed the substrate for VAs. Meanwhile, the increase of myocardium ischemia due to diminution of capillary distribution density among cardiomyocytes severely impacted systolic and diastolic myocardial function (2, 20). The above pathological changes were shown as high signals on LGE imaging. Consequently, we confirmed that significant myocardial fibrosis and myocardial strain impairment were present in HCM patients with VAs.

Previous studies evaluated the correlation between fibrosis detected by LGE and histological findings of fibrosis in patients with HCM (21–23). The size of LGE was positively associated with major risk factors of SCD and VAs, although results diverged in the role of %LGE in HCM risk stratification (24–27). Our study showed that %LGE >5.35% was able to distinguish HCM with VAs, which was not in line with other studies. We speculated that it might related with different study population and outcome events.

We evaluated myocardial strain of HCM by CMR-TT. The result demonstrated that decreased GCS was a marker of VAs in HCM, differently from other studies, reporting that impaired GLS was correlated to VAs (14, 28, 29). It has been reported that the muscle fibers of the ventricular wall were divided into three layers: the outer oblique myocardium, the middle circumferential myocardium, and the inner longitudinal myocardium (30). GLS reflected the longitudinal deformation of the myocardium, which was mainly related to the length changes of the inner myocardial fibers, so impaired subendocardial myocardium led to reduced GLS (11). Nevertheless, myocardial hypertrophy and fibrosis mainly occurred in the middle layer of myocardium, which increased myocardial heterogeneity (31). These pathological abnormalities aggravated the possibility of ventricular arrhythmias and contributed to the reduction of myocardial strain, especially GCS, which was related to the contraction and relaxation of the middle layer of the myocardium (10, 31). Therefore, our results showed that GLS was not sensitive enough to detect VAs in HCM. Besides, Jalanko et al. (32) did not find any significant correlation between GLS and VAs in the multivariate model after adjusting some parameters in HCM. In contrast, we found that declined GCS could assist in identifying HCM with VAs, and there was a good correlation between GCS and %LGE. Moreover, ROC curves showed that the addition of GCS to %LGE could better distinguish VAs in HCM patients (AUC = 0.87). Also, CMR-TT could easily detect radial, circumferential, and longitudinal strains based on the cine views. There was excellent reproducibility and stability of GCS (ICC > 0.9) between inter- and intra-observers in this study. The fact that it occurred in this small group of patients reinforced the importance of CMR-TT in the management of HCM.

### Clinical Implications

Myocardial strain detected by CMR-TT was helpful for early prediction of myocardial damage in HCM. GCS and %LGE were reliable and independent predictors for VAs in HCM patients. Reduced GCS may have potential value to identify HCM patients at risk of VAs, for whom LGE imaging quality is not enough to make a good diagnosis.

### Limitation

Several limitations were encountered during our study. First of all, the small number of patients from a single medical center might limit the interpretation of the results. Second, most enrolled HCM patients had present LGE. The main reason was that the vast majority of patients were transferred to our hospital from other centers after some clinical manifestations. Third, CMR images were obtained from two different kinds of scanners, which might affect myocardial strain measurements. Fourth, mapping technique combined with LGE might be more accurate to detect the presence and the extent of fibrosis in HCM, which could be evaluated in the future studies.

## Conclusion

In conclusion, myocardial strain parameters explored based on CMR-TT were helpful for early detection of myocardial damage in HCM patients. GCS and %LGE were strong and independent predictors for VAs in HCM. There was a correlation between myocardial strain and fibrosis detected by LGE. Reduced GCS may have incremental value to identify HCM patients with VAs.

## Data Availability Statement

The raw data supporting the conclusions of this article will be made available by the authors, without undue reservation.

## Ethics Statement

The studies involving human participants were reviewed and approved by Medical Ethics Committee of Sir Run Run Shaw Hospital, Zhejiang University School of Medicine. The ethics committee waived the requirement of written informed consent for participation.

## Author Contributions

CP and HH conceived and designed this study. CP and JF conducted the data collection and image analysis for this study. SL, CH, and DG contributed to the revision. PM, OC, and HH contributed to the language revision. CP drafted the manuscript. CP, YW, and HH did the proof work during the manuscript review. All authors listed have made a substantial, direct and intellectual contribution to the work, and approved it for publication.

## Conflict of Interest

The authors declare that the research was conducted in the absence of any commercial or financial relationships that could be construed as a potential conflict of interest.

## Abbreviations

ACEI: angiotensin converting enzyme inhibitor
ARB: angiotensin receptor blocker
BSA: body mass index
CMR: cardiac magnetic resonance
GCS: global circumferential strain
GCSDr: global circumferential strain of diastolic rate
GLS: global longitudinal strain
GLSDr: global longitudinal strain of diastolic rate
GRS: global radial strain
GRSDr: global radial strain of diastolic rate
HCM: hypertrophic cardiomyopathy
ICD: implantable cardioverter-defibrillator
LAD-AP: left atrial anteroposterior diameter
LGE: late gadolinium enhancement
LVEDVI: left ventricular end-diastolic volume index
LVEF: left ventricular ejection fraction
LVESVI: left ventricular end-systolic volume index
LVMI: left ventricular mass index
LVOT: left ventricular outflow tract
MWT: maximum wall thickness
SCD: sudden cardiac death
SVI: stroke volume index
VA: ventricular arrhythmia.
