# Supplementary material for: Global Circumferential Strain by Cardiac Magnetic Resonance Tissue Tracking Associated With Ventricular Arrhythmias in Hypertrophic Cardiomyopathy Patients
Source: Front Cardiovasc Med. 2021 May 28;8:670361. doi: 10.3389/fcvm.2021.670361 (PMC8193949; doi:10.3389/fcvm.2021.670361)
Supplement: Supplementary file 1 [file Data_Sheet_1.docx]

***Supplementary material***

**Supplementary Table 1. Myocardial strain in HCM patients with and without LGE**

|  | **HCM with LGE (78)** | **HCM without LGE (15)** | ***P*-value** |
| --- | --- | --- | --- |
| GRS (%) | 21.28 (16.86, 34.08) | 27.96 (24.45, 37.36) | 0.06 |
| GCS (%) | -16.49 (-19.82, -13.56) | -18.96 (-19.86, -17.40) | 0.08 |
| GLS (%) | -8.36 (-11.22, -6.65) | -10.47 (-12.12, -8.34) | 0.07 |
| GRSDr (1/s) | -1.29 (-2.19, -0.86) | -1.41 (-2.05, -1.26) | 0.32 |
| GCSDr (1/s) | 0.84 (0.58, 1.14) | 0.91 (0.72, 1.05) | 0.69 |
| GLSDr (1/s) | 0.50 (0.40, 0.63) | 0.59 (0.47, 0.65) | 0.23 |

Abbreviations: GCS, global circumferential strain; GCSDr, global circumferential strain of diastolic rate; GLS, global longitudinal strain; GLSDr, global longitudinal strain of diastolic rate; GRS, global radial strain; GRSDr, global radial strain of diastolic rate

**Supplementary Table 2. Analysis regarding the association of myocardial strain with LGE in HCM patients**

|  | ***r*** | ***P*-value** |
| --- | --- | --- |
| GRS (%) | -0.38 | <0.001 |
| GCS (%) | 0.51 | <0.001 |
| GLS (%) | 0.34 | 0.001 |
| GRSDr (1/s) | 0.18 | 0.09 |
| GCSDr (1/s) | -0.31 | <0.01 |
| GLSDr (1/s) | -0.19 | 0.07 |

Abbreviations: seeSupplementary Table 1

**Supplementary Table 3. Intra- and inter-observer reproducibility for LGE and myocardial strain measurements**

|  | **Intra-observer** | **Inter-observer** |
| --- | --- | --- |
|  | **ICC (95% CI)** | **ICC (95% CI)** |
| %LGE | 0.914 (0.702, 0.978) | 0.880 (0.599, 0.969) |
| GRS (%) | 0.653 (0.392, 0.817) | 0.728 (0.507, 0.860) |
| GCS (%) | 0.935 (0.870, 0.969) | 0.933 (0.865, 0.967) |
| GLS (%) | 0.865 (0.728, 0.934) | 0.899 (0.799, 0.950) |
| GRSDr (1/s) | 0.638 (0.370, 0.809) | 0.703 (0.468, 0.846) |
| GCSDr (1/s) | 0.667 (0.411, 0.826) | 0.655 (0.396, 0.818) |
| GLSDr (1/s) | 0.899 (0.801, 0.951) | 0.881 (0.765, 0.941) |

Abbreviations: CI, confidence interval; ICC, intraclass correlation coefficient; LGE, late gadolinium enhancement; others, seeSupplementary Table 1
